# Linderung der Blasenauslassobstruktion und Verbesserung der Symptome nach medikamentösen und chirurgischen Therapien bei Symptomen des unteren Harntrakts bei Benignen Prostatasyndrom

**DOI:** 10.1007/s00120-025-02572-y

**Published:** 2025-03-26

**Authors:** Lukas Koneval, Karl Georg Sommer, Laila Schneidewind

**Affiliations:** 1 Universitätsklinik für Urologie, Inselspital Bern, Freiburgstr. 37, 3010 Bern, Schweiz; 2 UroEvidence@Deutsche Gesellschaft für Urologie, Berlin, Deutschland

## Orginalpublikation

Creta M, Russo GI, Bhojani N, Drake MJ, Gratzke C, Peyronnet B, Roehrborn C, Tikkinen KAO, Cornu JN, Fusco F (2024) Bladder Outlet Obstruction Relief and Symptom Improvement Following Medical and Surgical Therapies for Lower Urinary Tract Symptoms Suggestive of Benign Prostatic Hyperplasia: A Systematic Review. Eur Urol 86(4):315–326. 10.1016/j.eururo.2024.04.031. Epub 2024 May 14. PMID: 38749852.

## Übersetzung

### Hintergrund und Zielsetzung.

Der symptomatische Nutzen und die urodynamische Linderung der Obstruktion stellen wesentliche Ergebnisse der Therapie von Symptomen des unteren Harntraktes (LUTS) im Zusammenhang mit benigner Prostatahyperplasie (LUTS/BPH) dar. Wir haben die Evidenz aus Studien zusammengefasst, die gleichzeitig Veränderungen in der Symptomschwere und in invasiven urodynamischen Messungen der Obstruktion nach medikamentösen und chirurgischen Therapien für LUTS/BPH untersuchen.

### Methoden.

Wir führten im Juni 2023 eine systematische Übersichtsarbeit durch und durchsuchten die Datenbanken PubMed, Scopus und Web of Science.

### Wichtigste Ergebnisse und Limitationen.

Wir identifizierten 29 Publikationen, darunter 14 Studien (872 Patienten) zu medikamentösen Therapien und 15 Studien (851 Patienten) zu chirurgischen Therapien. Die durchschnittliche prozentuale Verbesserung des International Prostate Symptom Score (IPSS) lag zwischen −2,5 % und 56,3 % nach medikamentöser Therapie und zwischen 35,1 % und 82,1 % nach chirurgischer Therapie. Die entsprechende durchschnittliche prozentuale Verbesserung des Bladder Outlet Obstruction Index (BOOI) reichte von 7,8–53,5 % bei medikamentöser Therapie und von 22,4–138,6 % bei chirurgischer Therapie. Unter den chirurgischen Verfahren zeigte die Holmiumlaserenukleation der Prostata (HoLEP) eine der höchsten Verbesserungen im IPSS sowie die stärkste Reduktion des BOOI**.**

### Schlussfolgerung und klinische Implikationen.

Insgesamt zeigt die verfügbare Evidenz, dass ausgeprägtere symptomatische Verbesserungen bei Behandlungen mit einer stärkeren desobstruktiven Wirkung beobachtet werden. Im Einzelnen weisen Patienten, die sich einer chirurgischen Behandlung unterziehen, größere Verbesserungen des IPSS und des BOOI auf als Patienten, die eine medikamentöse Therapie erhalten.

## Kommentar

Die systematische Übersicht von Creta et al. (2024) bietet eine aktuelle Synthese zur vergleichenden Wirksamkeit medikamentöser und chirurgischer Therapien der BPH in Bezug auf die Linderung der BOO und der LUTS. Die Methodik und Ergebnisse dieser Untersuchung liefern wertvolle Einblicke in die Optimierung patientenzentrierter Behandlungsstrategien und betonen die signifikante Symptomlinderung sowie urodynamische Verbesserung insbesondere durch die HoLEP.

Die Autoren führten eine systematische Übersicht der Literatur zwischen 2000 und 2023 durch und identifizierten 29 relevante Studien, die sowohl die Schwere der Symptome als auch invasive urodynamische Ergebnisse nach medikamentöser und chirurgischer Therapie von LUTS/BPH berichteten. Dieser methodische Ansatz stellt sicher, dass moderne Behandlungsmodalitäten berücksichtigt und die Entwicklung der BPH-Therapie in den letzten Jahrzehnten reflektiert wird. Die Methodik der Übersichtsarbeit folgt anerkannten wissenschaftlichen Standards wie PRISMA, PICOS und einer Risikoanalyse für Bias. Die Registrierung im PROSPERO-Register stellt ebenfalls ein Qualitätskriterium dar. Allerdings müssen Einschränkungen wie Heterogenität der Patientencharakteristika (z. B. urodynamische Merkmale sowie Behandlungsdauer und Nachbeobachtungszeiträume) und unterschiedliche Studiendesigns berücksichtigt werden. Weiterhin sind die Daten relativ aktuell. So identifizierte eine aktuelle Suche in Medline via PubMed bei Anwendung gleicher Query 278 Einträge (zum Zeitpunkt der Erfassung der Übersichtsarbeit waren es 220 Einträge).

Im Folgenden werden die zentralen Ergebnisse der systematischen Übersichtsarbeit zusammengefasst:

### Wirksamkeit chirurgischer vs. medikamentöser Therapien.

- Die Studie zeigt, dass chirurgische Eingriffe in Bezug auf symptomatische und urodynamische Ergebnisse deutlich überlegen sind.
- Die durchschnittliche prozentuale Verbesserung des IPSS lag zwischen 35,1 % und 82,1 % für chirurgische Therapien und zwischen −2,5 % und 56,3 % für medikamentöse Therapien.
- Der BOO-Index verbesserte sich bei chirurgischen Eingriffen signifikanter (22,4–138,6 %) als bei medikamentöser Behandlung (7,8–53,5 %).

### HoLEP als überlegene chirurgische Option.

- Unter den chirurgischen Verfahren zeigte HoLEP die größte Reduktion des BOO und eine substanzielle Verbesserung des IPSS, was seine Rolle als Goldstandard in der BPH-Behandlung unterstreicht.
- HoLEP bietet im Langzeitvergleich bessere funktionelle Ergebnisse und eine geringere Notwendigkeit für erneute Eingriffe als alternative chirurgische Verfahren zur BPH-Behandlung.

### Einschränkungen medikamentöser Therapien und Placeboeffekt.

- Die Studie hebt den Placeboeffekt in medikamentösen Studien hervor, insbesondere im Hinblick auf die subjektive Symptomverbesserung.
- Alpha-Blocker wie Tamsulosin und Silodosin führten zwar zu einer Reduktion des BOO, boten jedoch geringere Symptomlinderung im Vergleich zu chirurgischen Verfahren.

Die Ergebnisse dieser Untersuchung stehen im Einklang mit früheren Studien, die chirurgische und medikamentöse Behandlungen von LUTS/BPH verglichen. Die medikamentöse Therapie ist weniger wirksam als invasive Verfahren, insbesondere in Bezug auf Symptomverbesserung und Langzeiteffekt. Sie eignet sich v. a. für frühe Stadien der BPH oder für Patienten, die keinen Eingriff durchführen lassen können [1]. Eine kombinierte pharmakologische Therapie kann den Bedarf an chirurgischen Eingriffen bei Patienten mit größerem Prostatavolumen oder erhöhtem Progressionsrisiko verringern [2]. Die Entscheidung für eine Behandlung sollte individuell anhand der Symptomschwere, Prostatagröße, Patientenwünschen und möglicher Komplikationen getroffen werden. Chirurgische Eingriffe bieten bei ausgeprägter Obstruktion oder starker Symptomatik Vorteile [3].

Die medikamentöse Therapie eignet sich für Patienten mit milden bis moderaten LUTS ohne schwere Obstruktion. Eine chirurgische Intervention sollte bei hohem BOO (> 40), großer Prostata (> 80 ml) oder rezidivierender Harnverhaltung bevorzugt werden. Die HoLEP bietet langfristige Symptomlinderung mit geringer Reoperationsrate und zeigt sich vielen invasiven Verfahren überlegen [4]. Patienten mit Hochdruck‑/Niedrigflussmuster profitieren am meisten von der Chirurgie. Die urodynamische Untersuchung kann mit hoher Sicherheit zwischen BOO und Detrusorunteraktivität oder einer Kombination beider unterscheiden. Patienten mit schwereren Symptomen und niedrigerem Q_max_ profitierten am meisten von einer invasiven Therapie. Die urodynamische Untersuchung kann zusätzliche Informationen liefern, beeinflusst jedoch selten die Therapieentscheidung, außer bei Patienten mit Q_max_ > 15 ml/s, bei denen sie verborgene Obstruktionen besser aufdecken konnte. Die urodynamische Untersuchung kann insbesondere bei folgenden Patienten von Nutzen sein [5]: Patienten mit einer Vorgeschichte erfolgloser Behandlung und bei Patienten mit atypischen oder komplexen Beschwerden/Symptomen.

Die Übersichtsarbeit stellt die Hypothese auf, dass die Entfernung größerer Mengen adenomatösen Gewebes bei der HoLEP zu einer stärkeren urodynamischen Wirksamkeit führen könnte. Ein sinnvoller Vergleich besteht mit der offenen Adenomenukleation, die ebenfalls große Adenomvolumina entfernt. Naspro et al. zeigten, dass beide Verfahren bei großen Prostatadrüsen (> 70 g) signifikante urodynamische und funktionelle Verbesserungen erzielten [6]. Es traten keine signifikanten Unterschiede bei Q_max_, Restharnvolumen oder BOO nach 1, 3, 12 und 24 Monaten auf. Kuntz et al. (2008) bestätigten diese Ergebnisse auch bei noch größeren Adenomvolumina [7].

Insgesamt weist die Übersichtsarbeit mehrere Limitationen auf. Ein zentrales Problem ist die fehlende Standardisierung der urodynamischen Messungen, wodurch direkte Vergleiche zwischen den Studien erschwert werden. Zudem sind die Studienpopulationen heterogen, da unterschiedliche Ausgangsmerkmale vorliegen und einige Studien auch nicht-obstruierte Patienten einschließen, was die Ergebnisse potenziell verfälscht. Die geringe Anzahl an randomisierten kontrollierten Studien (RCT) und die oft kleinen Stichprobengrößen mindern die statistische Aussagekraft. Zudem waren nur 3 Studien placebokontrolliert, was die Abgrenzung zwischen echten pharmakologischen Effekten und Placeboreaktionen erschwert. Ein weiteres Problem stellt die häufig kurze Nachbeobachtungszeit dar, die Langzeitdaten zur Krankheitsprogression oder zur Notwendigkeit einer erneuten Behandlung unzureichend abdeckt. Schließlich besteht eine Einschränkung bei der Symptombewertung, da der häufig verwendete IPSS nicht immer mit den urodynamischen Befunden übereinstimmt und insbesondere Speichersymptome wie Drangsymptomatik oder Nykturie nicht adäquat erfasst, da dieser primär zur Bewertung obstruktiver Symptome (schwacher Harnstrahl, unvollständige Blasenentleerung) entwickelt wurde. Von den sieben Fragen des IPSS beziehen sich lediglich zwei auf Speichersymptome. Dies führt dazu, dass die Bewertung von Obstruktionssymptomen im Fokus steht, während Speichersymptome im Vergleich dazu weniger berücksichtigt werden.

Die Autoren der Übersichtsarbeit betonen, dass die Studiengrößen für minimalinvasive Therapien (MIT) oft klein waren, was die Wahrscheinlichkeit verringert, statistisch signifikante Unterschiede zu erkennen. Zudem fehlen teils Kontrollgruppen und randomisierte Designs. Laut Franco et al. (2021) zeigen die konvektive Radiofrequenz-Wasserdampf-Therapie (CRFWVT), der Prostataurethrallift (PUL), die Prostataarterienembolisation (PAE), das temporär implantierbare Nitinol-Device (TIND) und die transurethrale Mikrowellenthermotherapie (TUMT) signifikante Verbesserungen der Symptome gegenüber Placebo. Besonders PUL und PAE erzielen eine ähnliche Symptomlinderung wie die transurethrale Resektion der Prostata (TURP) bei geringerer Nebenwirkungsrate [8]. MIT bieten eine Alternative für Patienten, die eine weniger invasive Therapie bevorzugen. Die CRFWVT ermöglicht nachhaltige Symptomlinderung, verbessert den Harnfluss und beeinträchtigt die Sexualfunktion nicht [9]. Klare Kriterien für den Wechsel von einer pharmakologischen zu einer chirurgischen Therapie bei LUTS/BPH sind nicht definiert. Studien zeigen, dass Faktoren wie hohe Symptombelastung, unzureichendes Ansprechen auf Medikamente, Therapieadhärenz und Patientenpräferenzen die Entscheidung beeinflussen [1]. Ein chirurgischer Eingriff wird meist dann erwogen, wenn die medikamentöse Behandlung nicht ausreicht, die Symptome fortschreiten oder Komplikationen wie Blasensteine, therapieresistente Harnverhaltung, rezidivierende Infektionen, Überlaufinkontinenz oder Makrohämaturie auftreten. Leitlinien sollten Empfehlungen geben, wann ein Wechsel zur chirurgischen Therapie sinnvoll ist, bevor Komplikationen entstehen.

Die Pathophysiologie der Linderung von Symptomen des unteren Harntrakts ist aufgrund ihrer multifaktoriellen Natur umstritten. LUTS entstehen durch ein Zusammenspiel von Blasenauslassobstruktion, Blasenfunktionsstörung, neurogenen Faktoren und vaskulären Veränderungen, das zwischen Patienten variiert. Eine Herausforderung besteht in der Diskrepanz zwischen Symptomverbesserung und urodynamischen Veränderungen, da der IPSS nicht immer mit der BOO-Reduktion übereinstimmt. Faktoren wie Detrusorüberaktivität und veränderte afferente Signalwege tragen ebenfalls zu LUTS bei. Zudem beeinflussen nicht-obstruktive Komponenten wie Blasenumbau und Entzündungen die Persistenz der Symptome trotz Behandlung [5].

## Fazit für die Praxis

- Die Behandlung von Symptomen des unteren Harntraktes/benigner Prostatahyperplasie (LUTS/BPH) sollte individuell erfolgen, basierend auf: Symptomschwere, Patientenpräferenzen und dem Risiko für Komplikationen.
- Chirurgische Verfahren erzielen stärkere Verbesserungen der Symptome und der urodynamischen Parameter als medikamentöse Therapien.
- In schweren Fällen: Frühzeitige Beratung über chirurgische Optionen bei unzureichendem Ansprechen auf Medikamente.
- Minimalinvasive Verfahren sind eine vielversprechende Alternative für Patienten, die eine weniger invasive Behandlung wünschen.
